# Separable Sustained and Selective Attention Factors Are Apparent in 5-Year-Old Children

**DOI:** 10.1371/journal.pone.0082843

**Published:** 2013-12-20

**Authors:** Mette Underbjerg, Melanie S. George, Poul Thorsen, Ulrik S. Kesmodel, Erik L. Mortensen, Tom Manly

**Affiliations:** 1 Department of Public Health, Section of Epidemiology, Aarhus University, Aarhus, Denmark; 2 Children’s Neurocenter at Vejlefjord Rehabilitation Center, Stouby, Denmark; 3 MRC Cognition and Brain Sciences Unit, Cambridge, United Kingdom; 4 Department of Obstetrics and Gynaecology, Lillebaelt Hospital, Kolding, Denmark; 5 Department of Obstetrics and Gynaecology, Aarhus University Hospital, Aarhus, Denmark; 6 Institute of Public Health and Center for Healthy Aging, University of Copenhagen, Copenhagen, Denmark; Baycrest Hospital, Canada

## Abstract

In adults and older children, evidence consistent with relative separation between selective and sustained attention, superimposed upon generally positive inter-test correlations, has been reported. Here we examine whether this pattern is detectable in 5-year-old children from the healthy population. A new test battery (TEA-Ch^J^) was adapted from measures previously used with adults and older children and administered to 172 5-year-olds. Test-retest reliability was assessed in 60 children. Ninety-eight percent of the children managed to complete all measures. Discrimination of visual and auditory stimuli were good. In a factor analysis, the two TEA-Ch^J^ selective attention tasks (one visual, one auditory) loaded onto a common factor and diverged from the two sustained attention tasks (one auditory, one motor), which shared a common loading on the second factor. This pattern, which suggests that the tests are indeed sensitive to underlying attentional capacities, was supported by the relationships between the TEA-Ch^J^ factors and Test of Everyday Attention for Children subtests in the older children in the sample. It is possible to gain convincing *performance-*based estimates of attention at the age of 5 with the results reflecting a similar factor structure to that obtained in older children and adults. The results are discussed in light of contemporary models of attention function. Given the potential advantages of early intervention for attention difficulties, the findings are of clinical as well as theoretical interest.

## Introduction

In adults, attention processes have been primarily linked with extensive fronto-parietal networks [1,2,3]. The relatively late mylination of the prefrontal cortex [4], the role of genetic polymorphisms in determining distinct cognitive phenotypes [5] and the ubiquity of attentional problems across a range of childhood disorders have led to considerable interest in when, and in what form, these capacities emerge and develop. There are important discussions about whether and how the developing brain probabilistically ‘evolves’ towards a relatively more fixed adult modular structure. Influential accounts within this framework stress the potential importance of basic capacities, such as attention, in mediating this process [6]. Observational rating scales are important tools in this field. In addition, there is a need for performance-based measures that can support more precise operational definitions. Scerif et al. [7], for example, delineated distinct patterns of errors in young children with Fragile X and Williams syndrome, not apparent simply through observation, using a computerised visual search task. 

A particular issue is whether attention is usefully conceived as a unitary capacity, a set of relatively distinct processes, or an epiphenomena emerging from the interactions between brain systems without a separable neural basis. In an influential review, Posner and Petersen [2] argued from adult neuropsychological and functional imaging evidence that there were indeed distinct attentional networks vulnerable to selective damage. Further, that systems involved in selection (e.g. finding a visual target among distractors) diverged from those involved in sustained attention or alertness (e.g. maintaining a readiness to respond over a long interval) and spatial orienting. However, subsequent work has raised questions about how absolute such distinctions may be. Duncan and Owen’s influential meta-analysis of imaging studies [3] found that a wide variety of attentionally demanding tasks recruited *common* regions in frontal and parietal cortices (specifically, the posterior inferior frontal sulcus, the anterior insula/frontal operculum, the dorsal anterior cingulated and cortex in and surrounding the intraparietal sulcus). From this and other evidence (e.g. 8), it has been argued that these regions form a general, “multiple demand” or “global worskspace” network, flexibly adapting to focus processing on goal-relevant material [9,10]. It has been further suggested that, as a consequence of this central role, individual differences in the efficiency of this system may account for the generally positive correlations across almost all cognitive tasks [11]. 

Returning to Posner and Petersen’s position, however, it is important to emphasize that some separation between attention processes does not imply absolute divergence. Whatever the overlap, if there are processes that are disproportionately involved in one or other form of attention, interesting and clinically relevant differences may emerge. With the role of the global network in mind, carefully controlled studies are indeed reporting some specificity in attentional functions (e.g. 12). Similarly, reviewing the area in terms of potential phenotypic variations, Fan et al. [5] cite specific effects of differential neurotransmitter modulators on alerting, attentional orienting and target selection functions.

The above suggests a model in which diverse attentionally demanding tasks draw on common processes (leading to generally positive inter-task correlations) but which can also have specific features (leading to distinct superimposed clusters). This is precisely the pattern that emerged from a factor analysis on the normative data of the adult battery, the Test of Everyday Attention (TEA [13].) Here, performance on two timed visual search tasks and the color-word Stroop, loaded onto a common selective attention factor and diverged from two sustained attention tasks (a slow tone counting and vigilance level measure). Measures from the TEA were adapted to form the Test of Everyday Attention for Children (TEA-Ch [14]). Again, amid general inter-test correlation, the model based on separable selective and sustained attention factors formed a good fit to data from 293 children from the typically developing population. That the fit was not significantly stronger in the older (11-16), compared with the youngest (6-11) half of the sample suggested that these distinctions, evident in adulthood, were detectable in younger children. However, due to sample size in the relevant age-band, it remained unclear whether these patterns were entirely apparent by six.

There are good reasons why such patterns may be missed. The performance of the youngest children on the TEA-Ch, in addition to being less competent, showed markedly greater between-subject variability than that of older children. Such variance could result from variability in maturational rate at this age (which, in principle, would leave patterns of distinct attentional systems detectable), but also from extraneous influences such as variable test comprehension, motor development, motivation and compliance. In short, there is a risk that noise would overwhelm the attention signals in young children. Here we examined whether separable behavioral signatures of selective and sustained attention, similar to those seen in older children and adults, were detectable in a large group of 5-year-old children. 

Beginning with the TEA-Ch as our model, we aimed to reduce the influence of non-attentional factors by shortening the battery and minimizing memory and comprehension demands. To facilitate interpretation of the factor structure in terms of underlying process rather than incidental details of the tasks we varied the modality and response characteristics of the tests putatively assessing each factor. Selective attention tests included, for example, a cancellation task requiring a motor response, a visual search task requiring a present/absent verbal response and an auditory target detection task. Similarly, the sustained attention tests consisted of an auditory counting task and a slow-motor production task. Details of the tests and how they differed from the TEA-Ch are provided in the methods section. The tests were presented to 172 5-year-olds from the general population as a series games in a colorful comic. 

In summary, the aim of this study was to examine whether patterns consistent with distinct sustained and selective attention processes, present in adults and older children, were apparent in a factor analysis on data from a large group of 5-year old children from the healthy population. Interpretation of that analysis relies on a number of conditions being met. Specifically, evidence was required that children understood what was being asked of them, that they were generally motivated to perform well and that they were able to perform the tasks to some extent – i.e. whether performance-based assessment of attention was practicable *at all* in this age-group. Additional questions included the test-retest reliability of the new measures and their relationship to measures of general ability and the TEA-Ch. 

## Materials and Methods

### Participants

The study was approved by the Aarhus Regional Ethics Committee. Information and consent forms were distributed to all parents, caretakers or guardians of five-year-old children in 32 kindergartens in Aarhus County, Denmark. Written, informed consent was provided by parents, caretakers or guardians as appropriate on behalf of the children. Exclusion criteria were: Severe developmental disorders (e.g. autism, Down’s syndrome), severely impaired hearing or vision, or inability to speak Danish. Between 41 and 47 children were recruited in each of four 3-month age bands, yielding results from 172 children in total (see table 1). The relative rates of right- and left-handers were 161 and 11 (6.40%) respectively. 

**Table 1:** Participants in the four age bands.

Age band	years:months:days	Boys	Girls	Total
1	5:0:0 – 5:2:30	27	20	47
2	5:3:0 – 5:5:30	18	24	42
3	5:6:0 – 5:8:30	26	16	42
4	5:9:0 – 5:11:30	19	22	41
Total		90	82	172

### Measures

The TEA-Ch^J^ was an English-Danish collaboration. The original English manual was translated and back-translated by the authors and two bilingual consultants. The subtests were as follows;

#### TEA-Ch^J^: Balloon Hunt

**Figure 1:**
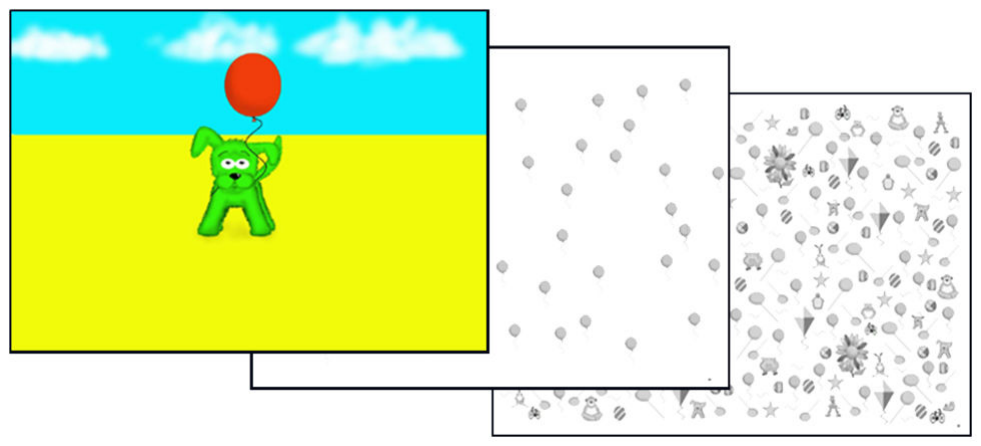
Balloon Hunt: The story in the stimulus book and the accompanying cancellation sheets showing the balloon targets with and without distractors.

In the original TEA-Ch Sky Search subtest, children were asked to search through series of pairs of spaceships arrayed in rows and columns, looking for and marking as quickly as possible those pairs where both ships were identical. This was completed under two conditions, one in which distractors were present (high attention load) and one in which only targets were presented (low attention load). The key variables were the time to self-reported completion in the two conditions. In the TEA-Ch^J^ adaptation (Balloon Hunt) a number of modifications were applied. Firstly, to make the task conceptually simpler, targets were defined by a common shape (identical balloons) rather than conjunction. Secondly, the rows and columns of Sky Search arguably afford systematic search strategies that were adopted by some but not all children. To reduce this affordance the 48 targets in each Balloon Hunt task were evenly but quasi-randomly distributed across the sheets. Another issue in interpreting Sky Search was whether slow performance reflected poor selection or an over-cautious style in which the sheet was repeatedly checked for missed targets before self-reported completion. To reduce this possibility, in Balloon Hunt 15-second time-limits were imposed for each sheet (Balloon Hunt 1 with no distractors, Balloon Hunt 2 with 116 distractors) – much less time than would be required to find all of the targets. This also had the merit of reducing overall testing time. In the comic the children were first introduced to the main dog character, told of his love of balloons and that he needed help to find as many balloons as possible within a short-time (see Figure 1). The task was then demonstrated to the children using a practice sheet and then their practice performance observed to ensure comprehension and ability to discriminate between targets and distractors. Further demonstrations/practice was given as needed before the two test items were administered. The number of targets and non-targets marked within the time-limit was recorded.

Since the development of the TEA-Ch evidence has grown that some children can show quite substantial lateralised visual biases, for example, missing a disproportionate number of targets on the left side of cancellation sheets (e.g. 15,16,17,18,19,20). Because the short time-limits on the first two Balloon Hunt conditions would preclude examination of spatial bias, a 3^rd^ cancellation sheet (Balloon Hunt 3) was included which included targets and distractors, had no time limit and was scored according to the number of omissions in each quadrant of the sheet.

#### TEA-Ch^J^: Barking

Most operational definitions of sustained attention emphasise performance decrements over long tasks. However, significant decrements can be difficult to observe over practicable test durations and the decrement model may be insensitive to fluctuations in attention that occur over shorter time-scales [21]. An alternative approach is to ask participants to keep a count of stimuli presented at a tediously slow rate – inattention at any point during the stream being likely to lead to an incorrect total [22]. Variants of slow tone counting procedures appeared in the TEA and TEA-Ch. However, for young children there is an increased risk that one is measuring counting ability rather than attention. Accordingly, in the TEA-Ch^J^ adaptation, (Barking), we both limited the number of stimuli to be counted to 6 and incorporated a control for counting skill by using a mix of fast and slow paced items. If a child was able to correctly count the same number of stimuli at a fast but not slow rate, interpretation in terms of sustained attention would be supported.

In the test, the children were told that they had found so many balloons that, in attempting to hold on to them all, the dog had been lifted into the air and was barking to gain assistance. They were asked to keep a count of how many barks occurred in each item of the test. The first practice item consisted of 3 barks presented at a slow pace to familiarize children with the idea that there may be long silent intervals and that a total should not be given until the end. In the second practice the barks were presented at a relatively fast pace to assess the ability to count to 6. Only if performance on both was adequate were the subsequent 10 items administered. Between 2 and 6 barks were presented in each, with four of the items having short inter-stimulus intervals (2-3 seconds) and the remainder using intervals of between 2 and 10 seconds. Whilst it was not suggested as a strategy, the children were allowed to count aloud but were discouraged from using fingers or other aids. The test was presented via speakers set to a comfortable level. The score was the total number of items correct/10.

#### TEA-Ch^J^: Draw-a-Line

**Figure 2:**
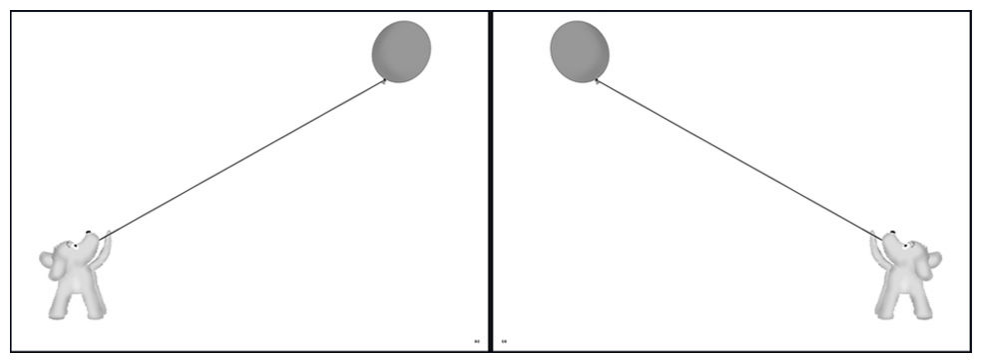
Response sheets for draw-a line.

A second sustained attention measure in the TEA-Ch was a vigilance level task (Code Transmission) that, at 10 minutes, contributed significantly to the total length of the battery. In the TEA-Ch^J^, we substituted the relatively brief and very different Draw-a-Line subtest. In the story, the dog attempts to tether a balloon in high winds. The children were asked to help strengthen the rope, making it thicker. In a practice, the children were given an A4 landscape sheet showing the dog gripping the rope (a 192 mm line) and were asked to trace *as slowly as possible* without deviating, stopping or lifting the felt pen. Once it was clear that these constraints were understood, the test item was administered. The practice and test sheets were identical but mirror reversed, allowing administrators to equate task ergonomics for left or right handed children by switching presentation order (see Figure 2). If children deviated from the line, stopped or lifted their pens during test performance, they were reminded of the instructions but allowed to continue. If repeated rule breaks occurred, administrators were given scope either to try again with a new sheet or abandon the measure. The measure was the time taken to completely trace over the line. The notion, as with similar tasks [23], is that this unusual goal must be kept actively in mind. If children forget – or simply become impatient with the delay – their final score will be lower. Clearly such tasks may also be sensitive to differences in motor control.

#### TEA-Ch^J^: Hide-and-Seek-V (visual)

**Figure 3:**
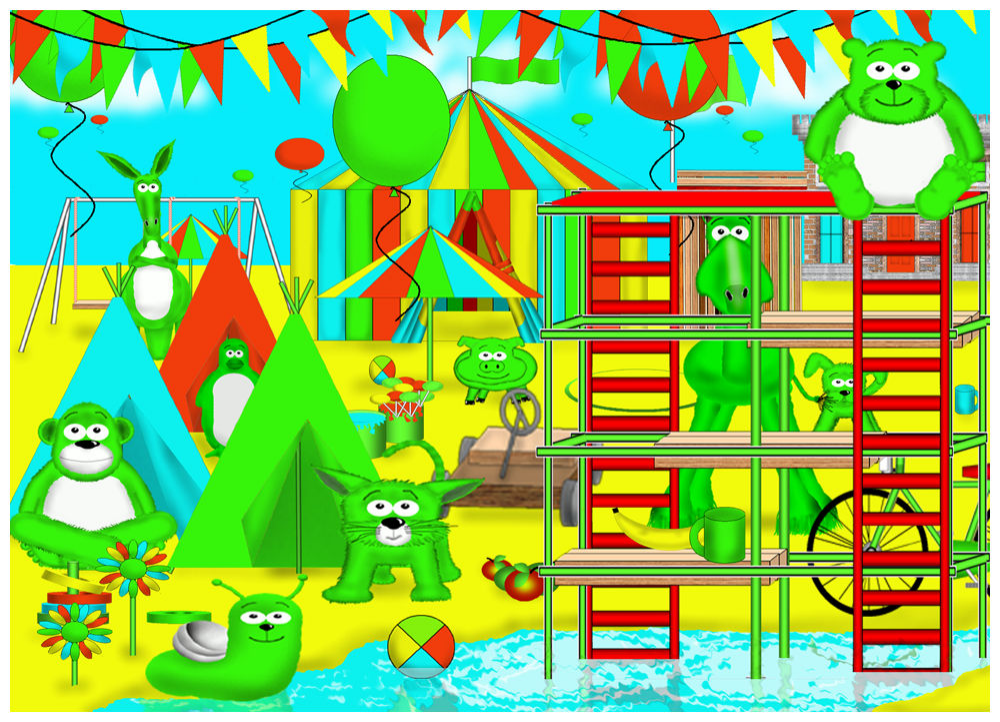
Hide-and-seek-V item.

In the TEA-Ch, both measures of visual selection (Sky Search and Map Mission) required speeded motor responses. In TEA-Ch^J^, a task requiring only a verbal response (Hide-and Seek-V) was included. The children were told that they were going to play hide-and-seek with the dog and that they needed to look carefully at each page and say “yes” as quickly as possible if he was there or “no” if they were certain that he was not. Following two practice items, eight test pages were administered (see Figure 3). To enhance sensitivity, the items varied in the number of distractors (drawings of other animals with similar colour and texture to the dog) and the prominence of the target. The dog was present on 4 items. The time between the page being exposed and an answer produced, and accuracy, were recorded.

#### TEA-Ch^J^: Hide-and-seek-A (auditory)

In the original TEA-Ch all selective attention measures were visual tasks. There were good reasons for this. There is greater complexity in audition (relative to vision) in separating effects of attention from physical and perceptual interference from competing sources. Children can show considerable within- and between- individual differences in hearing due to colds, infections etc. and it is difficult to standardise many clinical testing contexts in terms of background noise. However, given the likely importance of auditory attention in clinical descriptions of attention deficits this is clearly a limitation. Accordingly in TEA-Ch^J^ we developed the auditory selective attention task, Hide-and-Seek-A. The children were told that they must now listen for the dog. Following two practice items, 14 test items (10-second sound collage of animal and other non-verbal sounds presented at comfortable volume level) were administered. A countdown sequence of notes was used at the outset of each trial to allow administrators to synchronise their stopwatches with the last note. Timing was stopped when a response was given. Children were asked to say “yes” immediately following a bark but to wait for the end of the trial before saying “no”. The test was weighted in favour of target-present items (9/14) to maximise information from response times. 

To try and differentiate between inattention and hearing difficulties, 5 of the 14 items were direct repeats of an earlier item. The idea was that, if a child was correct on one but not another instance of the same item, transient inattention rather than frank difficulty hearing the target was a more likely account. 

The TEA-Ch included measures of executive control. These were not included in the TEA-Ch^J^. This was not because these functions (e.g. response inhibition, attentional switching) were thought to be absent in 5-year-olds, but rather because of the complexity of the instructions/tasks generally required may preclude clear interpretation in terms of these underlying capacities for this age group.

#### TEA-Ch: Score!

Following a practice used to establish comprehension and counting ability, the children were asked to count how many sounds had occurred in each of 10 items. Between 9 and 15 identical sounds were presented in each, separated by silent intervals of between 0.5 and 5 seconds. Piloting indicated that many 5-year-olds could not silently count and counting aloud was not penalised. For details of this and other TEA-Ch subtests see 14.

#### TEA-Ch: Sky search

Following practice, children were presented with a laminated A3 landscape orientation sheet and asked to find and mark all of the targets. The sheet showed a structured array of pairs of spaceships, with targets being defined by both ships in a pair being identical. In a subsequent condition, only the 20 target pairs were presented. A time-per-target measure (time from onset to self-reported completion divided by the number of targets detected) was calculated for each.

#### TEA-Ch: Map Mission

Here children were asked to search a colourful and visually noisy A3 Map for small target symbols. The score was the number of targets detected in a minute. 

#### Measure of General Abilities

Wechsler Preschool and Primary Scale of Intelligence – Revised[24] 

### Procedure

Following informed consent, individual testing took place in rooms that were relatively protected from visual and auditory distraction. The TEA-Ch^J^ and four WPPSI-R subtests were administered to all participants in one session lasting approximately 75 minutes (including breaks) in the following order: WPPSI-R Block design and Vocabulary; TEA-Ch^J^ Balloon Hunt, Barking, Draw-a-Line, Hide-and-seek-V and Hide-and-seek-A; WPPSI-R Object Assembly and Information. Seventy-six children in the oldest two bands were also administered 3 subtests from the TEA-Ch (in the order, Sky Search, Score! and Map Mission). 

Sixty children selected equally and at random from the youngest and oldest age bands were reassessed with TEA-Ch^J^ measures between 14 and 21 days (mean = 16.4 days) following their first assessment. Data on educational level was collected by asking parents to respond to a self-administered, postal questionnaire.

## Results

Before turning to the factor analysis, we first examine evidence relevant to its interpretation.

All but 3 children completed all of the TEA-Ch^J^ subtests and there was no child who did not manage to complete at least 3 of the measures. ‘Barking’ was abandoned on 3 occasions and the Hide-and-seek-A once. With short breaks, the TEA-Ch^J^ took approximately 30 minutes. 

**Figure 4:**
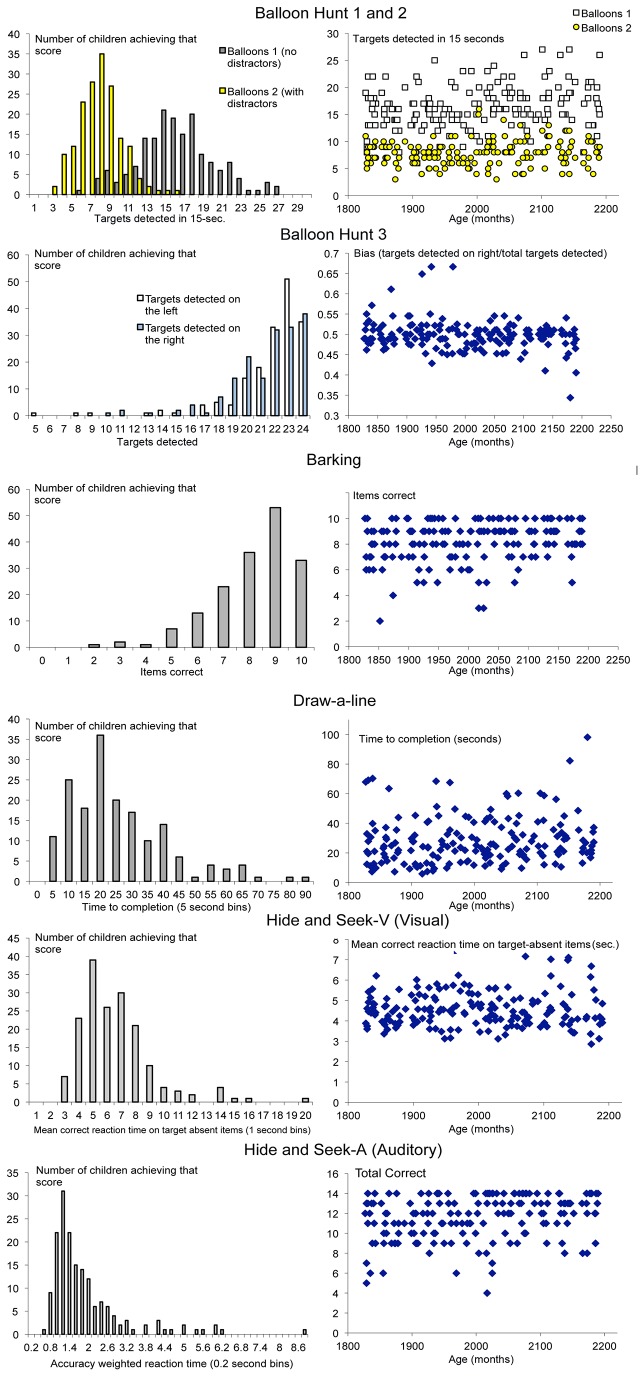
Score distributions from key TEA-Ch^J^ variables.

Details relating to score distributions, effects of age and differences between the boys and girls, relevant to the detailed discussion of each subtest below, are presented in table 2 and Figure 4. Within this 1-year range, performance-age correlations were generally modest and only reached statistical significance on 3 subtests. Although numerically girls outperformed boys on most measures, the difference was statistically significant only on Balloon Hunt 1. Parental education level (available for 93.6% of the participants; mean 14.66 years; SD 2.15) was not significantly correlated with children’s performance on any of the TEA-Ch^J^ subtests and was not considered in subsequent analyses.

**Table 2:** Distributions on key TEA-Ch^J^ variables and correlations with age.

Subtest	Girls mean (SD)	Boys mean (SD)	All mean (SD)	Median	Skewness	Kurtosis	Correlation with age
Balloons (targets detected in 1 and 2)	25.21* (5.45)	23.17 (5.10)	24.14 (5.35)	24	0.12	-0.16	0.27**
Spatial Bias (Balloons 3)	0.50 (0.04)	0.50 (0.04)	0.50 (0.04)	0.5	-1.06	7.19	-0.20**
Barking accuracy	8.20 (1.78)	8.15 (1.38)	8.17 (1.58)	9	-1.14	1.50	0.21** ^S^
Draw-a-line time	30.22 (15.23)	26.68 (16.46)	28.37 (15.94)	24.55	1.35	2.35	0.12
Hide-and-seek-V accuracy	7.65 (0.65)	7.54 (0.71)	7.59 (0.68)	8	-1.74	2.78	0.13^S^
Hide-and-seek-V time	5.94 (2.42)	6.29 (2.78)	6.12 (2.61)	5.83	1.83	5.13	0.10
Hide-and-seek-A accuracy	11.44 (2.29)	11.70 (2.12)	11.58 (2.20)	12	-0.90	0.41	0.28** ^S^
Hide-and-seek-A compound score	2.04 (1.69)	1.68 (1.14)	1.85 (1.14)	1.40	3.40	16.27	-0.18*

*p<0.05

^**^ p<0.01

s non-parametric Spearman’s rho used for scores with ceiling effects, otherwise Pearson’s r reported for correlations.

### Balloon Hunt

Children found a mean of 16.27 targets (SD 4.06) in 15 seconds in the Balloons Hunt 1 cancellation test. The addition of distractors in Balloon Hunt 2 had the expected attentional cost, with detection rates dropping to 7.87 (SD 2.28; repeated measures ANOVA (RMANOVA) F(1,171) = 822.26; p<0.001). No child found all of the targets in either condition (see Figure 4). Erroneous cancellation of distractors was rare (mean 0.15; SD 0.42).

On Balloon Hunt 3, which had no time-limit, mean detection rates were as expected near to ceiling at 43.08 (SD=4.83) and there was no statistically significant difference in detection rates between the two sides. (left target detection 21.69; SD 2.85; right 21.39; SD 2.65; F(1,170) = 2.29 p=0.132). However, this balanced picture may reflect two distinct underlying patterns. On some measures, adults and older children show a small but reliable attentional bias towards the left, termed pseudoneglect [25]. Here we used a bias score of targets detected on the right/total (i.e. scores above 0.5 represent right bias). As illustrated in Figure 4, there was indeed a small leftward bias in 91% of the sample (single sample t test on bias in those 157 children against 0.5 level performance t(156) = -2.30, P=0.02). This pattern was balanced in the overall left-right comparison by a small group of children who showed a more extreme rightward bias. Although age did not significantly correlate with overall target detection rates on the task (Pearson *r* (n =171) = -0.07, p = 0.34) it did significantly correlate with bias (r (n = 171) =-0.20, p = 0.008), with younger children tending to show more of a rightward bias. There was no indication that handedness influenced the pattern of results (left-handers mean bias score = 0.494, SD 0.023; right-handers 0.498, SD 0.038; F(1,169) = 0.073, p = 0.79), although there were relatively few left-handers (11) in the sample. It has previously been reported that poor sustained attention is a risk factor for abnormal rightward bias [15]. In this sample there was a significant correlation between bias and the Draw-a-Line measure from the sustained attention factor (r (172) = -0.19, P < 0.05); with children less able to maintain the goal (i.e. faster) showing higher rightward bias scores. The correlation remained when age was taken into account (partial correlation controlling for age r = -0.17, P = 0.03). Whilst the Barking subtest is prone to ceiling effects and did not significantly correlate with bias (Spearman’s rho = -0.38, P = 0.62), intriguingly 8/11 children with the lowest Barking scores (5/10 or fewer) showed a rightward bias.

### Barking

In the Barking test children were asked to keep count of a series of barks. Almost all children (91.7%) spontaneously counted aloud. As with the TEA-Ch equivalent, scores were skewed towards ceiling (mean 8.17/10; SD 1.58) with 19.53% scoring full marks. This suggests that the task was understood and that most children had the necessary counting ability. In addition, for 80% of the errors the response was out by 1, suggestive of transient inattention rather than guessing. As discussed, faster paced items were included as a control for counting difficulty. Accuracy was essentially at ceiling in these items (97.79% correct; SD 13.85) and significantly higher than the slow equivalents (73.67% SD 22.09; RMANOVA F(1,168) = 137.33, *P*<0.001). This suggests that errors were generally attributable to inattention rather than the counting or auditory demands of the task. 

### Draw-a-Line

All children managed to complete this task and, from their performance, all understood the instruction of tracing over the line as slowly as possible (mean 28.37 seconds; SD 15.94). Although 75% of the children completed the task within 35 seconds, the remainder were distributed in a tail stretching to 98 seconds. Imposing a 55 second cut-off would increase the discrimination of the measure for faster performers and reduce overall battery length. This would produce a mean performance of 27.38 seconds (SD 13.35).

### Hide-and-seek-V

Here, children were asked to report whether a visual target was absent or present in a series of pages. That no child had an error rate of more than 37.5%, and that 68.6% of the children were accurate on all items, suggests that the task was well understood and that misperception of the target/distractors was rare. 

As expected, correct ‘target absent’ responses took significantly longer (6.13 seconds; SD 2.61) than correct ‘target-present’ responses (3.50; SD 1.34; F(1,171) = 185.76 *P*<0.001). The error rates between these trial types did not vary (F(1,6) = 3.26, *P*=0.12). 

Given our expectations from pilot data, we were surprised how quickly the children produced correct responses on this task. This caused concern about the proportionate influence of the examiners’ own reaction times in stopping the watch. Accordingly, and contrary to original intentions, only correct response times on the target-absent items – in which more of the variance should be attributable to the children - were used in subsequent analyses.

### Hide-and-seek-A

In this test children listened to sound clips and reported whether a bark was present. That 90% of the sample scored 8/14 or higher suggests good comprehension and target discrimination. The intended difficulty gradient (the relative density of animal sound increased in later items) was confirmed (accuracy dropped from 6.27 (SD 1.09) for the first 7 items to 5.31(SD 1.59) for the remaining 7; RMANOVA F(1,171) = 66.22, *P*<0.001). 

Five items were repeated. A discrepancy occurred on at least one such item for 115 children (66%), that is they correctly identified the bark on one but not other presentation of the *identical* clip. Of the remaining children, 41 had no discrepancy because they were correct on all presentations of these items. There was no child who was incorrect on all repeated items. For the majority of children who made errors, therefore, at least one (in fact on average 1.5) of those errors was consistent with lapsing attention rather than poor hearing. This increases confidence that other errors on the test were similarly determined. The very low rate of premature responses (made by just 13 children and for them predominantly on only 1 of the 14 items) similarly suggests that auditory target discrimination was good. A combined measure was generated for this test in which the average correct reaction time was weighted by the proportion of items that were correct. 

### Factor Structure of the TEA-Ch^J^

A principal components analysis with varimax rotation on the 5 key TEA-Ch^J^ subtests produced two factors explaining 53% of the variance. Factor 1 loadings were highest for the selective attention tests (combined Balloon Hunt score = 0.80 and Hide-and-Seek-A 0.59). Factor 2 loadings were highest for the sustained attention measures (Barking 0.57 and Draw-a-Line 0.84). Hide-and-seek-V, perhaps predictably given the problems highlighted above, fared less well, loading equally on both factors (0.40 on factor 1 and 0.43 on factor 2). Raw correlations between the TEA-Ch^J^ measures are shown in table 3.

**Table 3:** Correlations between TEA-Ch^J^ subtests.

Subtest	Spatial bias (Balloons 3)	Hide-and-seek-V accuracy	Hide-and-seek-V time	Hide-and-seek-A compound score	Barking accuracy	Draw-a-line time
Balloons 1 & 2 targets detected	0.12	0.15* ^s^	0.06	-0.27**	0.21** ^s^	-0.02
Spatial Bias (balloons 3)		-0.05^s^	-0.09	-0.03	-0.04^s^	-0.19*
Hide-and-seek-V accuracy			0.16* ^s^	-0.14^s^	0.24** ^s^	0.07^s^
Hide-and-seek-V time				0.00	0.12^s^	0.08
Hide-and-seek A compound score					-0.26** ^s^	-0.13
Barking total correct						0.22**

^*^ p<0.05

^**^ p<0.01

s non-parametric Spearman’s rho used for scores with ceiling effects, otherwise Pearson’s r reported for correlations.

### TEA-Ch^J^ –TEA-Ch correlations

TEA-Ch subtests were only administered to the older (76) children and were not therefore included in the factor analysis. Examining the correlations with the TEA-Ch^J^ factor scores revealed that the TEA-Ch selective attention measures were significantly related to the TEA-Ch^J^ selection factor (Map Search r=0.53, p<0.01; Sky Search time r=-0.24, p<0.05, Sky Search motor control r= -0.43, p<0.01) but not to the TEA-Ch^J^ sustained factor (Map Search r=-0.05, Sky Search time r=0.03, Sky Search motor control r=0.01); and that the TEA-Ch sustained attention measure was significantly related to the TEA-Ch^J^ sustained factor (r=0.44, p<0.01); but not to the selection factor (r=0.08). 

### Test-retest reliability

Thirty children from age group 1 (18 boys and 12 girls) and 30 children from age group 4 (13 boys and 17 girls) were retested with the TEA-Ch^J^ between 14 and 21 days (mean 16.4 days) following their first assessment. As shown in table 4 test re-test correlations were generally adequate if moderate with the exception of Hide-and-seek-V. 

**Table 4:** Test-retest Pearson correlations (N=60).

Subtest	Mean (SD) at 1^st^ test	Mean (SD) at 2^nd^ test	Test-retest correlation
Balloon hunt (targets detected)	25.73 (4.96)	27.88 (6.27)	0.80**
Spatial bias (Balloon hunt 3)	0.50 (0.02)	0.50 (0.02)	-0.02
Barking accuracy	8.20 (1.55)	8.55 (1.28)	0.36** ^s^
Draw-a-line time	28.75 (16.52)	27.35 (17.86)	0.49**
Hide-and-seek-V accuracy	7.60 (0.72)	7.72 (0.56)	-0.07^s^
Hide-and-seek-V time	5.94 (2.31)	5.79 (2.18)	0.55***
Hide-and-seek-A accuracy	11.60 (1.95)	12.27 (1.92)	0.51** ^s^
Hide-and-seek-A compound score	1.49 (0.94)	1.33 (0.87)	0.51**

*p<0.05

^**^ p<0.01

s non-parametric Spearman’s rho used for scores with ceiling effects, otherwise Pearson’s r reported for correlations.

### Relationship to IQ

Based on Swedish norms the mean pro-rated WPPSI-R IQ of the sample was 108.4 (SD=12.93). Correlations between TEA-Ch^J^ and IQ are presented in table 5. With coefficients around the 0.3 level, correlations were statistically significant with the exception of the Balloons 3 spatial task (where ceiling scores were prevalent) and the Draw-a-Line test. 

**Table 5:** Pearson’s correlations between TEA-Ch^J^ key variables and subtests from TEA-Ch, IQ, and parental education level.

Subtest	TEA-Ch Sky Search (motor control)	TEA-Ch Sky Search (time-per-target)	TEA-Ch Map Mission	TEA-Ch Score!	Full scale IQ (prorated)	Parental education level
TEA-Ch^J^ Balloons 1 & 2 (targets detected)	-0.52***	-0.13	0.56***	-0.07	0.31***	0.07
Spatial bias (Balloons 3)	0.02	0.11	-0.18	0.12	-0.09	0.03
Barking accuracy	-0.01	-0.19	0.14	0.51***	0.22**	-0.08
Draw-a-line time	0.07	0.20	-0.06	0.22	-0.02	0.10
Hide-and-seek-V accuracy	-0.22	-0.25*	0.30**	0.25*	0.19*	0.11
Hide-and-seek-A compound score	0.07	-0.01	-0.17	-0.06	-0.16*	-0.06

^*^ p<0.05

^**^ p<0.01

^***^ p<0.001

## Discussion

The primary aim of this study was to examine whether separation between selective and sustained attention, apparent in adults and older children, was detectable in 5-year-olds. To this end, TEA-Ch battery (TEA-Ch) was simplified and shortened. Even the youngest children were able to tolerate the testing session, to understand what was required and to perform the tests. Although some ceiling effects were observed on the slow counting test, in other respects, the tests were sensitive across the ability range. 

Particular features of the tests were included to support or refute interpretation of performance variance principally in terms of attention. In the Barking subtest, for example, the children were less accurate on slow relative to fast paced counting items, suggesting that poor performance was not simply related to poor counting or hearing. Similarly, discrepant responses to repeated items in Hide-and-seek-A suggested that errors were generally due to attention lapses rather than poor hearing. Crucially for interpreting the factor analysis, tests designed to tap a common capacities differed quite markedly: The selective attention tests included a visuo-motor cancellation task and an auditory task. The sustained attention tests consisted of an auditory counting task (with some ceiling effect) and a motor task (with none). Common loading was therefore unlikely to arise as a result of modality, response type or irrelevant psychometric characteristics. 

Only one task proved problematic. In Hide-and-Seek-V, as discussed, we were surprised by the speed of the responses with the unfortunate consequence that the testers’ reaction times were probably a non-negligible component in the scores. Slowing the search by using more distractors/shared features and/or reducing the duration of presentation could improve the task.

With the exception of the Hide-and-Seek-V, the factor analysis was consistent with the hypothesised separation of selective and sustained measures superimposed upon generally positive inter-test correlations. The relationships between TEA-Ch performance and the factor scores in older children in the sample were also consistent with this picture. 

Considering overlap and differences between selective and sustained attention, let us first consider a well worked through example of selection in the visual system [26]. Objects and locations can be viewed as competing for representation. Aspects of this competition can be seen at many levels of the system. In infero-temporal cortex, for example, increased representation of one object will be at the expense of another [27]. In parietal cortex, attention to one spatial location will favour processing of a stimulus subsequently presented there at the cost of others [28]. What wins the competition at any given time will be influenced by inherent characteristics of stimuli or location (e.g. salience, movement, biological significance, spatial cues) but also by task-set or intention. It is this goal-directed attention that instruments such as the TEA-Ch^J^ aim to measure – the efficiency with which an arbitrary instruction held in working memory (e.g. “find balloons”) influences competition to produce fast, correct detections. Desimone & Duncan’s [26] key insight was that such intentional selection cannot occur only at the *end* of visual processing because the representation of relevant targets may already have been extinguished by irrelevant but salient rivals. Rather, task-relevant signals must influence the affray at *all* competitive levels of the system.

Attentional selection always has some duration. In that sense, all attention is *sustained* attention. However, the term is generally applied to rather specific conditions. These include measures in which participants must perform a repetitive task for long periods or remain engaged in a task despite extended intervals between relevant stimuli. As a consequence these tasks tend to be rather dull and vulnerable to competition from alternative tasks (daydreaming, thinking about when the session will end etc.). One useful way to conceptualise the difference between selective and sustained attention is therefore that, in the former, elements *within a single task-set* compete for representation whilst, in the latter, the competition is essentially between the entire task-set and more engaging rivals. The best candidates mediating stimulus selection within a task and competition between rival task-sets are ‘multiple-demand’ regions of the pre-frontal cortex. Studies have shown that, in contrast to say early visual neural populations that have rather fixed coding, a large proportion of prefrontal neurons show remarkable properties of rapidly coding *whatever is relevant to the task at hand* [29,30]. It has been argued that this activity biases competition in a goal directed fashion in regions engaged in early stimulus processing [10]. In terms of between task-set competition, prefrontal damage has been associated with increased distractibility, disorganisation, and problems in the voluntary maintenance of attention [22,31,32,33]. Multiple-demand efficiency may therefore form a good account of the general co-variance between many attentionally demanding tasks [11]. However, in a relatively engaging selective attention tasks of limited duration, it is much less likely that participants will ‘drift’ off-task (e.g. forget that they are looking for balloons). Here, differences in speed and efficiency of selection at multiple levels of processing would play a role and, in a factor analysis, we would expect a distinct cluster representing differences in these capacity limits.

The results here suggest that, long before full maturation, these distinctions are apparent in the performance of children. An important question, beyond the scope of the current paper, is whether such differential assessment is clinically useful in clarifying problems children may face in daily life and in better targeting interventions. While this has yet to be established in 5-year-olds, by inference from published studies using the TEA-Ch, this may well be the case [14,34,35,36,37,38,39]. 
